# Revisiting the pro-oxidant activity of copper: interplay of ascorbate, cysteine, and glutathione

**DOI:** 10.1093/mtomcs/mfad040

**Published:** 2023-06-23

**Authors:** Enrico Falcone, Francesco Stellato, Bertrand Vileno, Merwan Bouraguba, Vincent Lebrun, Marianne Ilbert, Silvia Morante, Peter Faller

**Affiliations:** Institut de Chimie (UMR 7177), University of Strasbourg—CNRS, 4 Rue Blaise Pascal, 67081 Strasbourg, France; Università di Roma Tor Vergata, Via della Ricerca Scientifica 1—00133 Roma, Italy; INFN, Sezione di Roma Tor Vergata, Via della Ricerca Scientifica 1—00133 Roma, Italy; Institut de Chimie (UMR 7177), University of Strasbourg—CNRS, 4 Rue Blaise Pascal, 67081 Strasbourg, France; Institut de Chimie (UMR 7177), University of Strasbourg—CNRS, 4 Rue Blaise Pascal, 67081 Strasbourg, France; Institut de Chimie (UMR 7177), University of Strasbourg—CNRS, 4 Rue Blaise Pascal, 67081 Strasbourg, France; Aix-Marseille Université, CNRS, BIP, UMR 7281, IMM, 31 Chemin Aiguier, 13009 Marseille, France; Università di Roma Tor Vergata, Via della Ricerca Scientifica 1—00133 Roma, Italy; INFN, Sezione di Roma Tor Vergata, Via della Ricerca Scientifica 1—00133 Roma, Italy; Institut de Chimie (UMR 7177), University of Strasbourg—CNRS, 4 Rue Blaise Pascal, 67081 Strasbourg, France; Institut Universitaire de France (IUF), 1 rue Descartes, 75231 Paris, France

**Keywords:** copper-thiolate clusters, copper toxicity, oxidative stress, reactive oxygen species, reducing agents, redox catalysis

## Abstract

Copper (Cu) is essential for most organisms, but it can be poisonous in excess, through mechanisms such as protein aggregation, trans-metallation, and oxidative stress. The latter could implicate the formation of potentially harmful reactive oxygen species (O_2_^•^^−^, H_2_O_2_, and HO^•^) via the redox cycling between Cu(II)/Cu(I) states in the presence of dioxygen and physiological reducing agents such as ascorbate (AscH), cysteine (Cys), and the tripeptide glutathione (GSH). Although the reactivity of Cu with these reductants has been previously investigated, the reactions taking place in a more physiologically relevant mixture of these biomolecules are not known. Hence, we report here on the reactivity of Cu with binary and ternary mixtures of AscH, Cys, and GSH. By measuring AscH and thiol oxidation, as well as HO^•^ formation, we show that Cu reacts preferentially with GSH and Cys, halting AscH oxidation and also HO^•^ release. This could be explained by the formation of Cu-thiolate clusters with both GSH and, as we first demonstrate here, Cys. Moreover, we observed a remarkable acceleration of Cu-catalyzed GSH oxidation in the presence of Cys. We provide evidence that both thiol-disulfide exchange and the generated H_2_O_2_ contribute to this effect. Based on these findings, we speculate that Cu-induced oxidative stress may be mainly driven by GSH depletion and/or protein disulfide formation rather than by HO^•^ and envision a synergistic effect of Cys on Cu toxicity.

## Graphical Abstract

**Graphical Abstract:**
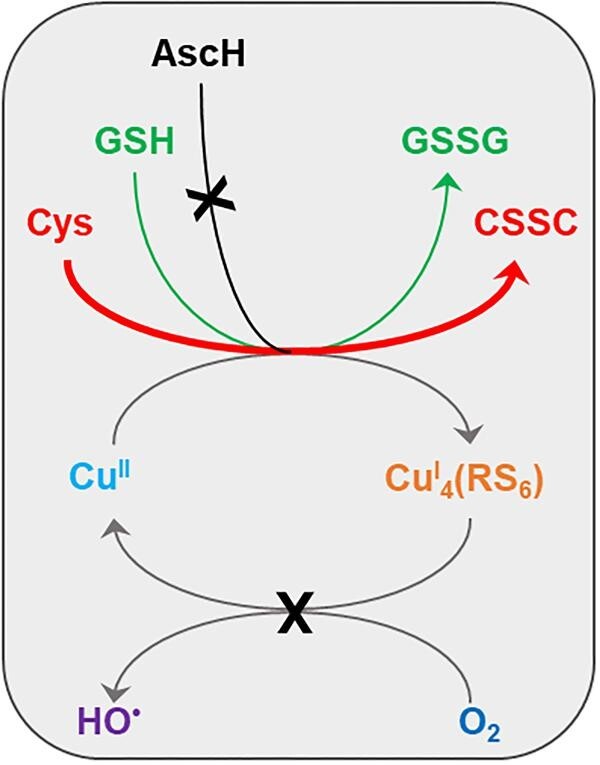
In a mixure of ascorbate (AscH), cysteine (Cys) and glutathione (GSH), Cu catalyzes the aerobic oxidation of Cys and GSH but not AscH, halting the release of hydroxyl radicals.

## Introduction

**Scheme 1:**
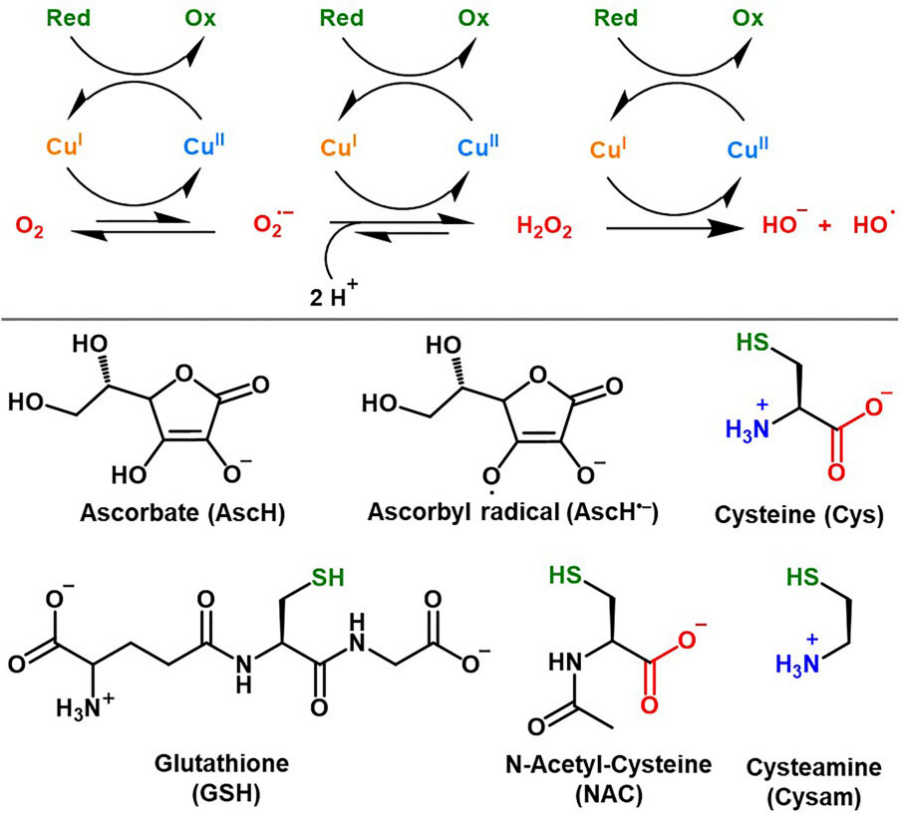
(*Top*) Mechanism of Cu-catalyzed ROS production in the presence of dioxygen and a reducing agent (Red), which is converted to its oxidized form (Ox). (*Bottom*) Possible reducing agents in cells are ascorbate (AscH), glutathione (GSH) and cysteine (Cys), which are converted into Ascorbyl radical (AscH^•−^), glutathione disulfide (GSSG) and cystine (CSSC), respectively. N-acetyl-cysteine (NAC) and cysteamine (Cysam) are Cys analogues.

Copper (Cu) is involved in fundamental biochemical processes (e.g. cellular respiration) and hence is an essential element for most organisms. In humans, Cu mostly serves as a redox cofactor of enzymes catalyzing the activation of oxygen (e.g. oxidases and monooxygenases) via its cycling between Cu^I^ and Cu^II^ redox states.^1^ To prevent undesired Cu redox activity outside the active sites of Cu enzymes, a set of extracellular carriers, membrane transporter, and cytosolic Cu-chaperones ensure a safe Cu transport in the body. Such transporters stabilize Cu in one of the possible redox states, notably Cu^II^ in the oxidizing extracellular milieu and Cu^I^ in the reducing intracellular environment.^2^ Nevertheless, their “Cu-buffering” capacity can be overcome in the case of Cu overload, eventually resulting in Cu toxicity. Along with the recently identified trans-metallation of Fe-S clusters and protein aggregation,^3–7^ the aerobic redox chemistry of labile (i.e. loosely bound) Cu, which can lead to the formation of potentially harmful reactive oxygen species (ROS, such as O_2_^•^^−^, H_2_O_2_, and HO^•^, Scheme 1) and oxidative stress, has been commonly considered accountable for Cu toxicity.^8^ Among ROS, HO^•^ radical is considered to be one of the most dangerous, due to its higher intrinsic reactivity and to the absence of specific scavenging systems, which instead exist for O_2_^•^^−^ (i.e. superoxide dismutase, SOD) and H_2_O_2_ (catalase).^9^ Cu-catalyzed ROS generation can be fueled by different physiological reducing agents (Scheme 1), such as ascorbate (AscH), glutathione (GSH), and cysteine (Cys).

AscH is present up to millimolar concentration in cells and is very competent in inducing Cu-catalyzed ROS formation in the test tube.^10,11^

GSH is the most abundant thiol in cells (1–10 mM), where it helps keep Cu in the reduced Cu^I^ state.^12^ In the test tube, GSH binds Cu^I^ in relatively redox-stable Cu-thiolate clusters, and in cells, it binds labile Cu in the case of overload.^13–15^ In contrast, Cys (Scheme 1), which reacts with Cu faster than GSH,^16^ is found at much lower concentrations (30–250 µM) and becomes toxic at higher levels.^17^

Although the behavior of Cu with each of the earlier-mentioned reductants has been investigated, little is known about the reactions taking place in a more physiologically relevant mixture of these biomolecules. Therefore, we explored the reactivity of Cu with binary and ternary mixtures of the earlier-mentioned biomolecules, with the aim to speculate about the possible mechanisms behind Cu-induced oxidative stress.

## Results and discussion

### Impact of thiols on Cu-catalyzed ascorbate oxidation and hydroxyl radical generation

**Fig. 1:**
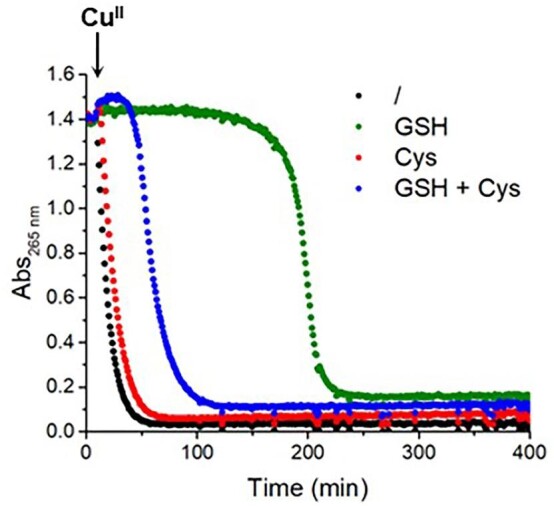
Effect of thiols on Cu^II^-catalyzed oxidation of AscH. Ascorbate oxidation in the presence of Cu^II^ only (black), Cu^II^ and GSH (green), Cu^II^ and Cys (red), Cu^II^, GSH and Cys (blue); conditions: [AscH] = 100 µM, [Cu^II^] = 10 µM, [GSH] = 100 µM, [Cys] = 100 µM, phosphate buffer 50 mM pH 7.4.

First, we assessed the effect of thiols on Cu-catalyzed aerobic AscH oxidation, which can be monitored photometrically via the decrease of its characteristic absorption at 265 nm. Unless otherwise stated, reactions were performed in open microplates, ensuring the availability of dioxygen (dissolved concentration of about 270 µM). In the absence of thiols, the addition of Cu^II^ promptly triggered the aerobic oxidation of AscH (Fig. 1), which is complete within ∼50 min. When GSH was present (at a concentration equal to AscH, 100 µM) before Cu addition, the onset of AscH oxidation appeared to be remarkably delayed (Fig. 1, green). We supposed, in agreement with the literature,^18^ that during such a lag phase Cu catalyzed the aerobic oxidation of GSH to glutathione disulfide (GSSG). Indeed, the quantification of GSH through the classical 5,5'-dithio-bis-(2-nitrobenzoic acid) (DTNB) assay (also known as Ellmann's test) showed that (in the absence of AscH) Cu^II^ was able to catalyze the aerobic oxidation of GSH in a timespan (∼180 min) similar to the lag phase observed in the AscH oxidation (Supplementary Fig. S1, green).^†^ Cys also delayed the onset of Cu-catalyzed AscH oxidation, despite to a much lesser extent (∼5 min) compared to GSH, which is coherent with the very much faster Cu^II^-catalyzed oxidation of Cys compared to GSH (Fig. 1 and Supplementary Fig. S1, red).^16^ Actually, such an inhibitory effect of thiols on Cu-catalyzed AscH oxidation has been long known,^18–22^ and can be interpreted considering that Cu^II^ is first reduced and coordinated by the thiols, where it is stabilized in the Cu^I^ state, and catalyzes their oxidation to disulfide by O_2_, while it catalyzes the oxidation of AscH only once little or no reduced thiols are present. Interestingly, when both Cys and GSH were present, the lag phase in AscH oxidation was much shorter than in the absence of Cys (Fig. 1, blue), suggesting that Cys accelerates GSH oxidation, as we also observed in the absence of AscH (Supplementary Fig. S1, blue). Remarkably, such acceleration is also observed with 10-fold less Cys (100 µM) than GSH (1 mM), which are in the range of physiological concentrations (Supplementary Fig. S2).

Since Cu-catalyzed aerobic oxidation of AscH and thiols is normally accompanied by the formation of ROS (Scheme 1), we also followed the HO^•^ production using coumarin-carboxylic acid (CCA), which forms the fluorescent 7-hydroxy-CCA (λ_ex_ = 390 nm, λ_em_ = 452 nm) upon reaction with HO^•^. Interestingly, no HO^•^ was detected as long as reduced thiols were present (Supplementary Fig. S3A). Noteworthy, the time delay observed for the onset of HO^•^ production fits that of the beginning of AscH oxidation. Moreover, although no HO^•^ was detected in the presence of Cys only (Supplementary Fig. S3B), the latter was oxidized too fast to significantly delay the HO^•^ production in the presence of AscH. Of note, the measurement of 4-hydroxy-2,2,6,6-tetramethylpiperidin-1-oxyl (TEMPOL) electron paramagnetic resonance (EPR) signal, which is quenched by reaction with HO^•^, also showed that no significant amount of HO^•^ is produced by Cu^II^ in the presence of thiols (Supplementary Fig. S3C).

### Spectroscopic evidence for the formation of Cu-Cys clusters

In order to explain why the Cu-catalyzed GSH oxidation by O_2_ does not generate HO^•^ radicals, it has been suggested that GSH-bound Cu^I^ reduces H_2_O_2_ to H_2_O, rather than HO^•^.^23^ This process is indeed plausible, especially in light of the currently known formation of multinuclear Cu*_x_*(GS)*_y_* clusters,^13^ where the proximity of several Cu ions could favor the occurrence of a two-electron reduction. Since Cu^II^ did not produce HO^•^ in the presence of Cys, we assessed whether Cu-thiolate clusters are also formed with Cys by means of X-ray absorption spectroscopy (XAS) and low-temperature luminescence.

**Fig. 2:**
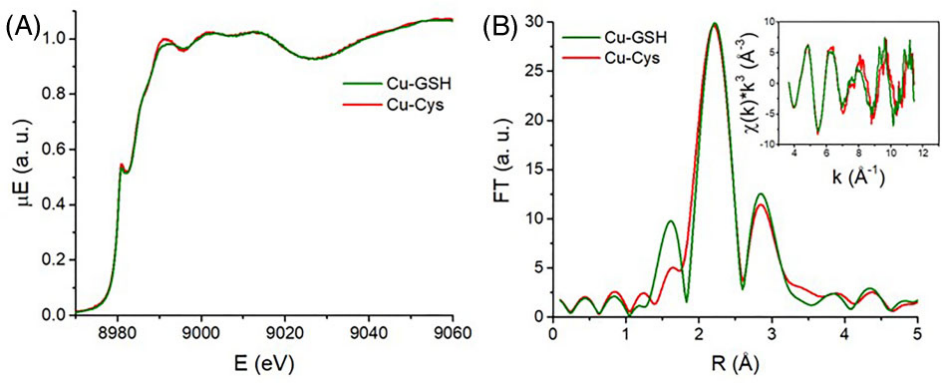
XANES spectra (A) and Fourier transforms (B) of the EXAFS (inset) spectra of Cu-GSH (green) and Cu-Cys (red) complexes. Conditions: [Cu^II^] = 1 mM, [GSH] = 10 mM, [Cys] = 10 mM, phosphate buffer 200 mM pH 7.4.

Both the Cu K-edge X-ray absorption near edge spectroscopy (XANES) and extended X-ray absorption fine structure (EXAFS) spectra (Fig. 2) of Cu-Cys and Cu-GSH samples are indistinguishable within the experimental error. Therefore, the Cu coordination in Cu-Cys is substantially identical to that in Cu-GSH, which forms mostly Cu_4_(GS)_6_ clusters.^13^ Indeed, the EXAFS data can be fitted by a model Cu_4_S_6_ cluster,^24,25^ in which each Cu atom is surrounded by three S atoms at about 2.3 Å and a “disordered” shell (i.e. with relatively high values of σ^2^, the mean square deviation of the distance) composed of three Cu atoms at about 2.8 Å (Supplementary Fig. S4 and Supplementary Table S1), although models with a different number of Cu scatterers cannot be ruled out solely based on EXAFS data.

**Fig. 3:**
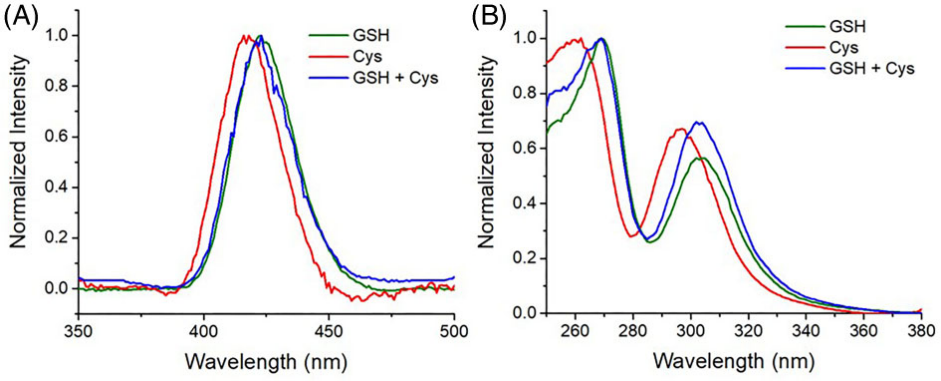
Low-temperature (77 K) luminescence emission (A) and excitation (B) spectra of Cu–thiols mixtures (GSH, green; Cys, red; GSH and Cys, blue). Conditions: [Cu^II^] = 100 µM, [GSH] = 1 mM, [Cys] = 1 mM, phosphate buffer 50 mM pH 7.4.

In light of the known luminescent emission of Cu_4_(GS)_6_ and Cu-metallothioneins clusters,^13,26^ we also recorded low-temperature (77 K) luminescence spectra of Cu-Cys and Cu-GSH. Interestingly, upon excitation at 310 nm, Cu-Cys exhibited a luminescent emission band at 418 nm, similar but slightly blue-shifted compared to the emission of Cu-GSH at 423 nm (Fig. 3A), attributed to tetranuclear Cu_4_(GS)_6_. Likewise, the excitation spectrum (λ_em_ = 310 nm) of Cu-Cys appears to be blue-shifted compared to that of Cu-GSH (Fig. 3B), in agreement with the absorption spectra reported in the literature.^13,27^ This further confirms that, similarly to GSH, Cu and Cys form tetranuclear Cu_4_(Cys)_6_ clusters. In addition, we measured the luminescence of a mixture of Cu, GSH and Cys, which showed emission and excitation spectra very similar to those of Cu-GSH clusters, suggesting that GSH has a higher affinity for Cu than Cys. Notwithstanding, the accelerating effect of Cys on GSH oxidation suggests that a minor portion of Cu-Cys or mixed Cu-GSH/Cys clusters exist.

### Insights into the mechanism of Cys-accelerated GSH oxidation

Previous reports have shown the acceleration of GSH oxidation by Cys in the absence of Cu or the presence of Cu, Zn-SOD, and the thiol-disulfide exchange reaction between Cys disulfide, CSSC, and GSH (Eq. 1) has been postulated as the mechanism.^28–30^


(1) \begin{equation*}2\,{\rm{GSH}} + {\rm{CSSC}}\rightarrow 2\,{\rm{Cys}} + {\rm{GSSG}}\end{equation*}

**Fig. 4:**
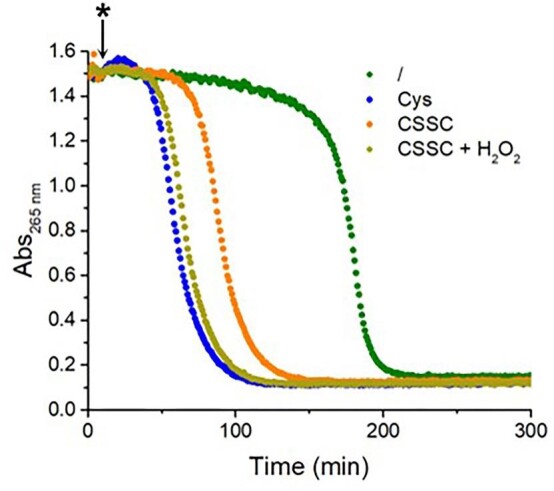
Effect of CSSC and H_2_O_2_ on the Cu^II^-catalyzed oxidation of AscH in the presence of GSH. Conditions: [AscH] = 100 µM, [Cu^II^] = 10 µM, [GSH] = 100 µM, [Cys] = 100 µM, [CSSC] = 50 µM, [H_2_O_2_] = 50 µM, phosphate buffer 50 mM pH 7.4. First, AscH and GSH were pre-mixed; then, at the time point indicated by *, the following compounds were added: Cu^II^ (green), Cu^II^ and Cys (blue), Cu^II^ and CSSC (orange), Cu^II^, CSSC and H_2_O_2_ (gold).

To assess such a hypothesis, we compared the kinetics of GSH oxidation by Cu^II^ in the presence of Cys and CSSC using the lag phase in AscH oxidation assay as a convenient read-out for the thiol oxidation kinetics. Indeed, if CSSC accelerates GSH oxidation simply via thiol-disulfide exchange, a shorter lag phase (of less than ∼5 min in our conditions, i.e. the time needed to completely oxidize Cys to CSSC) would be expected when directly adding the corresponding amount (0.5 equivalent, i.e. 50 µM) of CSSC, rather than Cys, to the mixture containing AscH, GSH, and Cu^II^. Actually, although CSSC accelerated GSH oxidation, the lag phase proved to be longer when 50 µM CSSC, rather than 100 µM Cys, was added (Fig. 4, orange), suggesting that the catalytic effect of Cys on GSH oxidation is not merely driven by the thiol-disulfide exchange between CSSC and GSH. Considering that H_2_O_2_ is also formed along Cys oxidation to CSSC,^31^ we explored the possibility that H_2_O_2_ also contributes to the acceleration of GSH oxidation. Indeed, the addition of 50 µM H_2_O_2_ together with 50 µM CSSC (Fig. 4, gold), decreased the lag phase more than CSSC alone, and notably to a very similar extent compared to 100 µM Cys (Fig. 4, blue). Therefore, along with the thiol-disulfide exchange previously suggested, the H_2_O_2_ produced during Cys oxidation by Cu^II^ seems to contribute to the acceleration of GSH oxidation. Interestingly, the Cu/Cys-catalyzed aerobic GSH oxidation could contribute to Cys toxicity and also exacerbate the toxicity of excess Cu in cells.

**Fig. 5:**
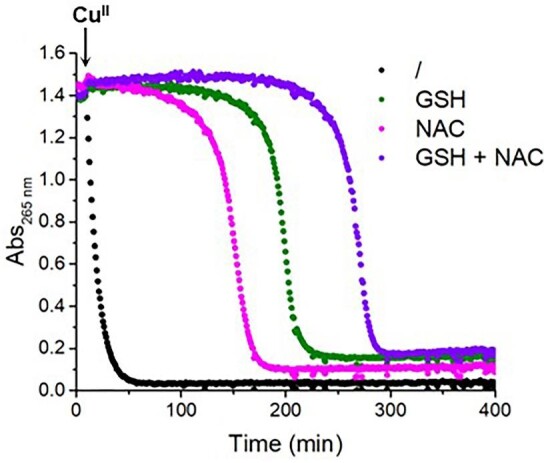
Effect of NAC on GSH oxidation. Ascorbate oxidation in the presence of Cu^II^ (black), Cu^II^ and GSH (green), Cu^II^ and NAC (magenta) or Cu^II^, GSH and NAC (violet); conditions: [AscH] = 100 µM, [Cu^II^] = 10 µM, [GSH] = 100 µM, [NAC] = 100 µM, phosphate buffer 50 mM pH 7.4.

To better understand the faster oxidation of Cys compared to GSH and its accelerating effect, we also examined the reactivity of its derivatives N-acetyl-cysteine (NAC, Scheme 1), which is commonly used as an antioxidant,^32^ and cysteamine (Cysam, Scheme 1), a simple aminothiol. Thus, we assessed the effect of NAC and Cysam on the GSH oxidation in the presence of Cu^II^ using the AscH oxidation assay (Fig. 5 and Supplementary Fig. S5). Cu^II^-catalyzed aerobic NAC oxidation also appeared to be faster than GSH oxidation (Fig. 5, magenta), but to a much lower extent than for Cys. Instead, Cysam reacted, at least, as fast as Cys (Supplementary Fig. S5). However, contrary to Cys and Cysam (Supplementary Fig. S5), NAC had little (if at all) impact on the rate of GSH oxidation (Fig. 5, violet). Such faster oxidation of Cys, Cysam, and NAC compared to GSH could be explained by their ability to chelate Cu^II^ in a bidentate fashion via the thiol group together with the amino (Cys and Cysam) or carboxylate (NAC) moiety (Scheme 1). Indeed, since the re-oxidation of Cu^I^ to Cu^II^ is considered the rate-limiting step of the Cu-catalyzed thiol oxidation,^16^ the stabilization of the Cu^II^ state by Cys, Cysam, and NAC can fasten the reaction. Besides, the negligible effect of NAC on the rate of GSH oxidation can be attributed to its higher pK_a_ (9.5) compared to Cys (8.3) and Cysam (8.2), which implies a much slower thiol-disulfide exchange.^33,34^

## Conclusions

Excess labile Cu is generally considered to be toxic owing, among other mechanisms, to the formation of ROS through O_2_ reduction by Cu^I^ ions. Hence, Cu-catalyzed ROS production depends primarily on dioxygen (whose level in cells can vary quite a lot, from normoxic, to hypoxic such as in cancer, down to zero in cells living anaerobically) and reducing agents. In cells, the most relevant Cu reductants are AscH, GSH, or Cys. In this study, we confirm that in a mixture of such biomolecules, oxidation of the two thiols occurs before that of AscH. Interestingly, the spectrophotometric measurement of AscH concentration at 265 nm results to be a convenient *in situ* readout of thiol oxidation. Importantly, and in line with the literature,^23^ Cu-catalyzed aerobic GSH/Cys oxidation is not accompanied by the release of harmful HO^•^ radicals, which are instead detected during Cu-catalyzed aerobic AscH oxidation. It is worth noting that this behavior is not an intrinsic feature of thiols, as HO^•^ can be detected when thiol oxidation is catalyzed by Cu-complexes.^35^ Hence, we speculate that such reactivity of thiols arises from the formation of Cu-thiolate clusters. Indeed, we showed that Cys also forms tetranuclear Cu-S clusters similar to the already known Cu_4_(GS)_6_.^13^ Interestingly, the proximity of several Cu ions in a multinuclear cluster could favor the two-electron reduction of H_2_O_2_ to H_2_O that was suggested by previous studies.^23^ Alternatively, HO^•^ radicals could be formed by Cu-GSH/Cys but react with the neighboring thiols of the cluster, preventing their release and detection by external probes (CCA or TEMPOL in this study). Hence, the potential role of Cu-thiolate clusters in preventing HO^•^ formation or release is worthy of further experimental and computational investigations. Besides, in analogy with previous reports,^28–30^ we observed that Cys, as well as Cysam but not NAC, accelerates Cu-catalyzed GSH oxidation, although Cu-GSH is the predominant species even in the presence of equimolar Cys. We also showed that GSH oxidation is accelerated even at physiologically relevant sub-stoichiometric Cys: GSH ratio of 1:10. Moreover, not only thiol-disulfide exchange but also oxidation via H_2_O_2_ contributes to the Cu-catalyzed acceleration of GSH oxidation by Cys.

From a biological perspective, our findings suggest that, in the case of Cu overload, oxidative stress arises, in the first instance, from aberrant disulfide, notably GSSG, formation, rather than HO^•^ production. It is indeed well established that excess Cu promotes GSH depletion, altering the GSH/GSSG balance.^15,36–38^ Although this impairs cellular redox homeostasis, excess Cu buffering by GSH helps to protect other potential Cu targets, such as protein thiols. Indeed, upon GSH depletion, Cys-containing proteins with high Cu-affinity (e.g. those containing the CXXC motif)^39^ could undergo Cu-catalyzed oxidation resulting in loss of function. Moreover, as recently demonstrated, Cu can also target protein thiols in compartments such as bacterial periplasm, where GSH concentration is lower.^40,41^ Furthermore, in light of the recent discovery of lipoylated proteins as preferential targets of Cu toxicity,^6^ the ability of Cu to catalyze lipoic acid (a dithiol) oxidation despite the presence of excess GSH remains to be assessed. Whether Cu-catalyzed protein disulfide formation is accompanied by HO^•^ formation is another important aspect that warrants future studies. Interestingly, the ability of Cu to oxidize and hence deplete Cys faster than GSH, could be one of the reasons why, during evolution, an N-protected thiol like GSH, rather than an aminothiol like Cys, was selected as the most abundant intracellular thiol: GSH represents a more resilient Cu buffering system than Cys, ensuring longer protection and survival in the case of Cu stress. Finally, it is noteworthy that, as it has been shown for Fe,^42^ high Cys levels may exacerbate the poisonous effects of excess Cu ions on cellular redox homeostasis, and hence the increase of Cys levels can be envisioned as a strategy to enhance the cytotoxic activity of Cu for therapeutic purposes.

## Materials and methods

### Stock solutions

Commercially available chemicals were used without further purification. All stock solutions were prepared in ultrapure water (ρ = 18.2 MΩ · cm^−1^). Cu^II^ stock solution was prepared by dissolving CuCl_2_ · 2H_2_O salt and its concentration was assessed by ultraviolet–visible spectroscopy absorption at 780 nm (ε_780_ = 12 M^−1^^ ^cm^−1^). A stock solution of the phosphate buffer (pH 7.4) was prepared by mixing KH_2_PO_4_ and K_2_HPO_4_ and adjusting the pH with a concentrated solution of NaOH. Solutions of sodium AscH, GSH, Cys, and NAC were freshly prepared before the experiments. Cysteamine stock solution was prepared as follows: Cysam hydrochloride powder was flushed with N_2_, then dissolved in 1 mM HCl thoroughly flushed with N_2_ and stored at -20°C. The concentration of Cysam was determined via the DTNB assay (see following text).

### Ascorbate oxidation assay

AscH oxidation was monitored by absorption at 265 nm on a CLARIOstar (BMG Labtech) plate reader inside an open 96-well microplate (Greiner) or in a closed cuvette using an Agilent Cary 60 spectrophotometer (Supplementary Fig. S2). After mixing AscH (100 µM) and thiols (100 µM each) in phosphate buffer (50 mM, pH 7.4), the signal was monitored for about 10 min to assure no AscH auto-oxidation was taking place. Then, Cu^II^ (10 µM) was added and the reaction was monitored over time.

### DTNB assay

Thiols oxidation was measured upon the reaction of reduced thiols with the Ellmann's reagent, DTNB, monitoring the formation of the TNB^2−^ product by absorption at 412 nm on a CLARIOstar plate reader inside a 96-well microplate (Greiner). After mixing Cu^II^ (10 µM) and thiols (100 µM each) in phosphate buffer (50 mM, pH 7.4), aliquots (25–50 µl) were taken at several time points and transferred to the assay mixture (final volume 100 µl) containing 100 µM DTNB and 1 mM EDTA in 50 mM TRIS buffer pH 8.2 (final concentration of thiol being 50 µM). Thiol concentration was calculated using ε_412_ = 14,150 M^−1^cm^−1^.

### CCA assay

The formation of 7-hydroxy-coumarin-3-carboxylic acid (7-OH-CCA) was monitored by fluorescence emission at 452 nm upon excitation at 390 nm on a CLARIOstar plate reader inside a 96-well microplate (Greiner). Cu^II^ (10 µM) was added to a solution containing CCA (500 µM) and AscH, thiols or their mixture (each at 100 µM) in phosphate buffer (50 mM, pH 7.4) and the reaction was monitored over time.

### EPR spin scavenging

EPR spin scavenging experiments were performed at room temperature (*T* = 295 ± 1 K) using an EMX-plus (Bruker Biospin GmbH, Germany) X-band EPR spectrometer equipped with a high sensitivity resonator (4119HS-W1, Bruker). Samples were introduced into glass capillaries (Hirschmann, 25 µl) sealed at both ends and rapidly transferred into the EPR cavity for measurement. The principal experimental parameters were microwave frequency of ∼9.8 GHz, microwave power of ∼4.5 mW, modulation amplitude 1 G, time constant of ∼5 ms, conversion time of ∼12.5 ms. A scan (sweeping time of ∼10 s) was then acquired every 17 s to obtain the kinetics of TEMPOL reduction over time. All spectra were best simulated and the resulting simulations were doubly integrated to relatively quantify the concentration of remaining TEMPOL [*I*/*I*_0_ = *I*(*t*)/*I*(*t* = 0)]. Data analysis and simulations based on experimental data were performed using Xenon (Bruker Biospin GmbH) and lab-made routines based on EasySpin Toolbox under Matlab (Mathworks) environment.^43^

### XAS

XAS data at the Cu K-edge were acquired at the BM30 beamline of the European Synchrotron Radiation Facility (ESRF—Grenoble, France). The beamline energy was calibrated using a metallic Cu foil by setting the position of the absorption edge (defined as the first maximum of the first derivative curve) to 8979 eV. Spectra were recorded in fluorescence mode using a 13-element solid-state Ge detector. In order to minimize X-ray-induced damage, the samples were kept at 10 K in a He cryostat throughout the measurements. The ATHENA software^44^ was used to normalize XANES data and to extract the EXAFS signal, which was obtained by cubic splines interpolation as implemented in the AUTOBKG algorithm.^45^ The quantitative analysis of the EXAFS spectra was performed using the EXCURV98 code.^46^

### Low-temperature luminescence

Low-temperature luminescence spectra were recorded using a FluoroMax Plus spectrofluorometer (Horiba Scientific) equipped with a cylindrical quartz Dewar filled with liquid nitrogen (at 77 K). A total of 500 µl samples were transferred to quartz tubes with 4 mm inner diameter and freeze-quenched into liquid nitrogen before their introduction in the Dewar.

## Supplementary Material

mfad040_Supplemental_File

## Data Availability

Data available on request.

## Footnotes

† The results show that GSH oxidation to GSSG is independent of the presence of AscH, indicating that AscH is not catalyzing GSH oxidation. Indeed, the steadiness of AscH could have been only apparent, and notably due to a fast reduction of the ascorbyl radical (AscH^•–^) by GSH.[47] This appears to be at most negligible in light of the very similar kinetics of thiols oxidation in the absence and in the presence of ascorbate.
