# Supplementary material for: Revisiting the pro-oxidant activity of copper: interplay of ascorbate, cysteine, and glutathione
Source: Metallomics. 2023 Jun 23;15(7):mfad040. doi: 10.1093/mtomcs/mfad040 (PMC10331802; doi:10.1093/mtomcs/mfad040)
Supplement: mfad040_Supplemental_File [file mfad040_supplemental_file.pdf]

Supplementary Material

Revisiting the pro-oxidant activity of copper: interplay of ascorbate, glutathione and cysteine

Enrico Falcone,[a*] Francesco Stellato,[b,c] Bertrand Vileno,[a] Merwan Bouraguba,[a] Vincent Lebrun,[a] Marianne Ilbert,[d] Silvia Morante[b,c] and Peter Faller[a,e]

[a] *Institut de Chimie (UMR 7177), University of Strasbourg – CNRS, 4 Rue Blaise Pascal, 67081 Strasbourg, France*
[b] *Università di Roma Tor Vergata, Via della Ricerca Scientifica 1 - 00133 Roma, Italy*
[c] *INFN, Sezione di Roma Tor Vergata, Via della Ricerca Scientifica 1 - 00133 Roma, Italy*
[d] *Aix-Marseille Université, CNRS, BIP, UMR 7281, IMM, 31 Chemin Aiguier, 13009 Marseille, France*
[e] *Institut Universitaire de France (IUF), 1 rue Descartes, 75231 Paris, France*

Contents

Figure S1. Cu^{II}-catalysed thiols oxidation S2

Figure S2. Effect of Cys on GSH oxidation at 1:10 Cys/GSH ratio S2

Figure S3. $HO^{•}$ generation in the presence of Cu^{II} and different reductants S2

Figure S4. Fit of EXAFS data S3

Table S1. EXAFS best-fit parameters S3

Figure S5. Effect of Cysam on GSH oxidation S3

[*] corresponding author
email: enrico.falcone@nottingham.ac.uk
current address: School of Chemistry, University of Nottingham, University Park, NG7 2RD Nottingham, UK

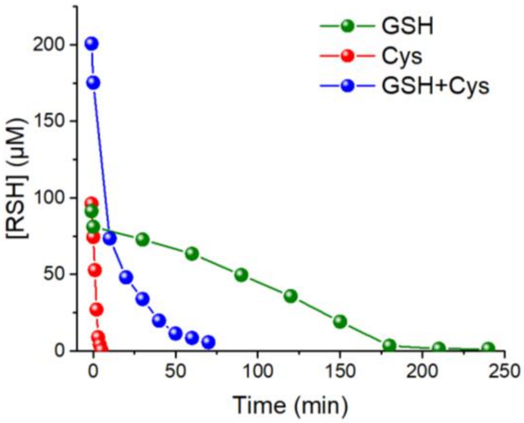

Figure S1. Cu^{II}-catalysed thiols (GSH, green; Cys, red; GSH and Cys, blue) oxidation measured via the DTNB (Ellmann's) assay. Conditions: [Cu^{II}] = 10 µM, [GSH] = 100 µM, [Cys] = 100 µM, phosphate buffer 50 mM pH 7.4.

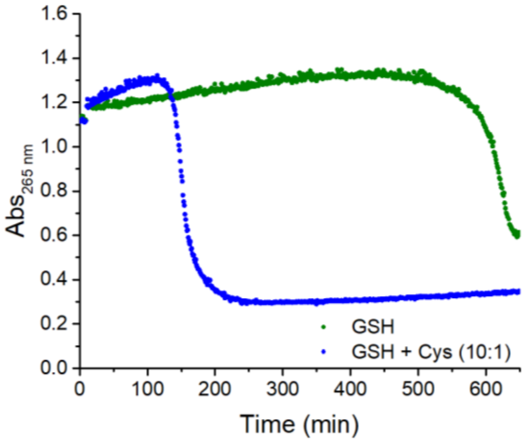

Figure S2. Effect of Cys on GSH oxidation at 1:10 Cys/GSH ratio. Ascorbate oxidation in the presence of Cu^{II} and GSH (green) or Cu^{II}, GSH and Cys (blue); conditions: [AscH] = 100 µM, [Cu^{II}] = 10 µM, [GSH] = 1 mM, [Cys] = 100 µM, phosphate buffer 50 mM pH 7.4. In order to prevent sample evaporation during the overnight incubation, the reaction was performed inside a closed microcuvette. Hence, the incomplete Ascorbate oxidation observed may be attributed to limited amount of O_2.

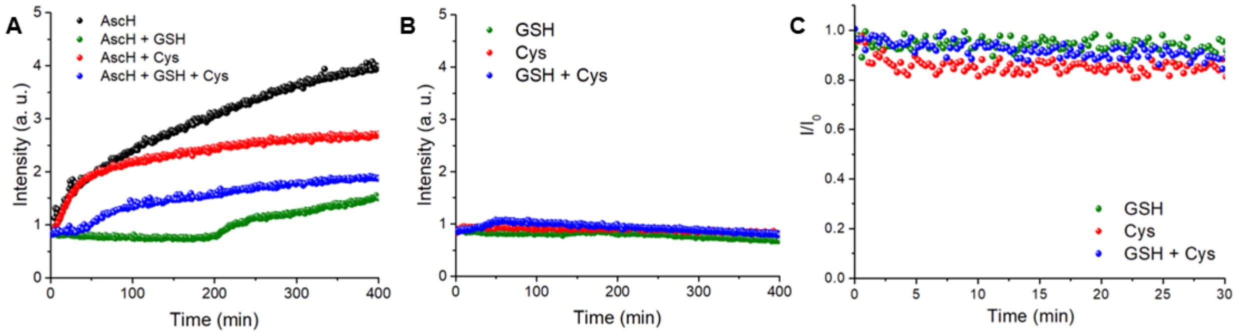

Figure S3. $HO^{\bullet}$ generation in the presence of Cu^{II} and different reductants (AscH, black; AscH and GSH, green; AscH and Cys, red; AscH, GSH and Cys, blue monitored via the fluorometric CCA assay (A, B) or EPR spin scavenging with TEMPOL (C). (A, B) conditions: [CCA] = 500 µM, [AscH] = 100 µM, [Cu^{II}] = 10 µM, [GSH] = 100 µM, [Cys] = 100 µM, phosphate buffer 50 mM pH 7.4. (C) conditions: [TEMPOL] = 20 µM, [Cu^{II}] = 10 µM, [GSH] = 100 µM, [Cys] = 100 µM, phosphate buffer 50 mM pH 7.4.

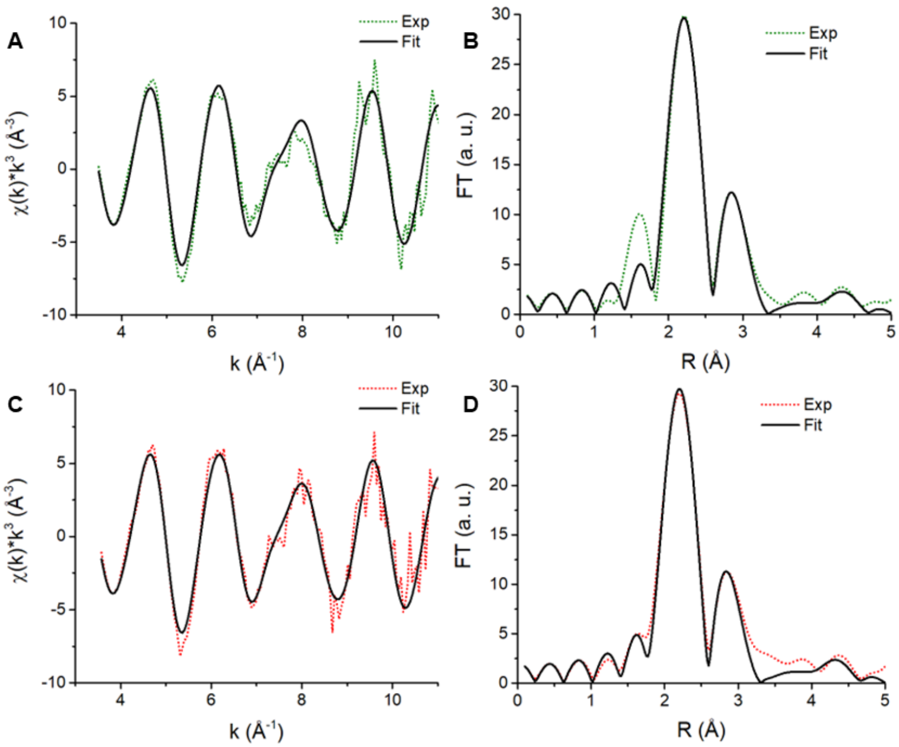

Figure S4. Experimental EXAFS data (dotted lines) and best-fit theoretical models (full lines) of Cu-GSH (A) and Cu-Cys (C) complexes and corresponding Fourier Transforms (B and D, respectively).

Table S1. EXAFS best-fit parameters for Cu-GSH and Cu-Cys complexes. The number of each type of backscattering atom (N), the distance between absorbing atom (i.e., Cu) and the backscattering atom (R), the mean square deviation in R (Debye−Waller factor; σ^2), and the energy offset to the threshold energy (ΔE_F) are given.

		Cu-GSH				Cu-Cys			
**Ligand**	**N**	**R [Å]**	**σ^2 [Å^2]**	**ΔE_F (eV)**	**R**	**R [Å]**	**σ^2 [Å^2]**	**ΔE_F (eV)**	**R**
S	3	2.28(1)	0.003(1)	-1.5(5)	26 %	2.27(1)	0.003(1)	-0.7(5)	22 %
Cu	3	2.74(1)	0.011(2)			2.73(1)	0.012(2)		

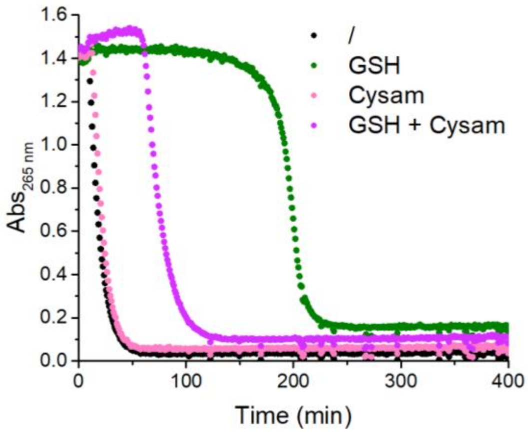

Figure S5. Effect of Cysam on GSH oxidation. Ascorbate oxidation in the presence of Cu^{II} (black), Cu^{II} and GSH (green), Cu^{II} and Cysam (pink) or Cu^{II}, GSH and NAC (purple); conditions: [AscH] = 100 µM, [Cu^{II}] = 10 µM, [GSH] = 100 µM, [Cysam] = 100 µM, phosphate buffer 50 mM pH 7.4.
